# Phosphorylation of NF2 at Serine-13 by MAP4K family kinases mediates pathological angiogenesis

**DOI:** 10.1093/procel/pwac005

**Published:** 2022-10-14

**Authors:** Mingyue Ma, Zhenxing Zhong, Yuwen Zhu, Yuan Gu, Ruxin Jin, Zhipeng Meng, Yu Wang, Fa-Xing Yu

**Affiliations:** Institute of Pediatrics, Children’s Hospital of Fudan University, and the Shanghai Key Laboratory of Medical Epigenetics, the International Co-laboratory of Medical Epigenetics and Metabolism, the State Key Laboratory of Genetic Engineering, Institutes of Biomedical Sciences, Shanghai Medical College, Fudan University, Shanghai 200032, China; Institute of Pediatrics, Children’s Hospital of Fudan University, and the Shanghai Key Laboratory of Medical Epigenetics, the International Co-laboratory of Medical Epigenetics and Metabolism, the State Key Laboratory of Genetic Engineering, Institutes of Biomedical Sciences, Shanghai Medical College, Fudan University, Shanghai 200032, China; Institute of Pediatrics, Children’s Hospital of Fudan University, and the Shanghai Key Laboratory of Medical Epigenetics, the International Co-laboratory of Medical Epigenetics and Metabolism, the State Key Laboratory of Genetic Engineering, Institutes of Biomedical Sciences, Shanghai Medical College, Fudan University, Shanghai 200032, China; Institute of Pediatrics, Children’s Hospital of Fudan University, and the Shanghai Key Laboratory of Medical Epigenetics, the International Co-laboratory of Medical Epigenetics and Metabolism, the State Key Laboratory of Genetic Engineering, Institutes of Biomedical Sciences, Shanghai Medical College, Fudan University, Shanghai 200032, China; Institute of Pediatrics, Children’s Hospital of Fudan University, and the Shanghai Key Laboratory of Medical Epigenetics, the International Co-laboratory of Medical Epigenetics and Metabolism, the State Key Laboratory of Genetic Engineering, Institutes of Biomedical Sciences, Shanghai Medical College, Fudan University, Shanghai 200032, China; Department of Molecular and Cellular Pharmacology and Sylvester Comprehensive Cancer Center, University of Miami Miller School of Medicine, Miami, FL 33136, USA; Institute of Pediatrics, Children’s Hospital of Fudan University, and the Shanghai Key Laboratory of Medical Epigenetics, the International Co-laboratory of Medical Epigenetics and Metabolism, the State Key Laboratory of Genetic Engineering, Institutes of Biomedical Sciences, Shanghai Medical College, Fudan University, Shanghai 200032, China; Institute of Pediatrics, Children’s Hospital of Fudan University, and the Shanghai Key Laboratory of Medical Epigenetics, the International Co-laboratory of Medical Epigenetics and Metabolism, the State Key Laboratory of Genetic Engineering, Institutes of Biomedical Sciences, Shanghai Medical College, Fudan University, Shanghai 200032, China

**Dear Editor,**

Angiogenesis is vital for the development and maintenance of functional organs, and also participates in diverse pathological processes, such as wound healing, oxygen tension, and tumorigenesis (Potente et al., 2011). Thus, understanding the molecular mechanisms responsible for angiogenesis has important clinical implications and may guide strategies for drug development.

The Hippo pathway is an evolutionary conserved signaling cascade that plays critical roles in development, tissue homeostasis, and tumorigenesis (Wang et al., 2017b). Yes-associated protein (YAP) and transcriptional coactivator with PDZ binding motif (TAZ), the two major downstream effectors of the Hippo pathway, are expressed in endothelial cells and required for angiogenesis. Conditional deletion of Yap/Taz in endothelial cells during mouse development results in severe vascular defects and embryonic lethality (Kim et al., 2017, Wang et al., 2017a), highlighting the importance of YAP/TAZ in angiogenesis and early development. Moreover, mitogen-activated protein kinase kinase kinase kinase 4 (MAP4K4), a Hippo-like kinase that acts as a repressor of YAP/TAZ, is involved in both developmental and pathological angiogenesis (Meng et al., 2015, Zheng et al., 2015). Mechanistically, MAP4K4 phosphorylates moesin, a FERM (F for 4.1 protein, E for ezrin, R for radixin, and M for moesin) domain-containing protein, to regulate focal adhesion dynamics, membrane retraction, and endothelial cell migration (Vitorino et al., 2015). How deficiency of YAP/TAZ and their negative regulator causes defects in angiogenesis remains unclear, but likely involves additional MAP4K4 substrates that participate in angiogenesis.

Neurofibromin 2 (NF2), also known as Merlin, is a key component of the Hippo pathway (Yu et al., 2015). While NF2 is known to be expressed in endothelial cells, its role in endothelial cell behavior has not been characterized. Similar to moesin, NF2 is a FERM domain-containing protein that serves as a membrane-cytoskeleton scaffold to mediate diverse signaling pathways (McClatchey and Giovannini 2005). Cells with NF2 deficiency form unstable cell–cell junctions (Lallemand et al., 2003, Wang et al., 2021), which are critical for migration of endothelial cells and formation of tubular structures. Thus, we speculate that NF2 may have a role in angiogenesis.

NF2 is phosphorylated at multiple sites (Fig. 1A), but the functional roles of most phosphorylation sites are unknown. To probe regulation of NF2 phosphorylation, we examined NF2 mobility in polyacrylamide gel electrophoresis in the presence of a phos-tag reagent, the latter was used to separate proteins according to their phosphorylation status. As shown in Fig. 1B, NF2 proteins were resolved into multiple bands, suggesting that NF2 was phosphorylated at multiple sites. We then purified ectopically expressed NF2 and performed phosphopeptide mapping by mass spectrometry analysis (Fig. 1C). In addition to Ser518 (S518) which has been studied previously, two other sites—Ser12 (S12) and Ser13 (S13)—were identified as phosphorylated sites (Fig. S1A). By alanine substitution analysis, phosphorylation events of NF2 were matched with different bands on phos-tag gels (Fig. 1D). Of note, a weak band corresponding to S13 phosphorylation was consistently observed (Fig. 1D). To further examine this phosphorylation site, we made a S13 phospho-specific antibody that sensitively detects S13 phosphorylation of ectopically expressed NF2 and immunoprecipitated endogenous NF2 (Figs. 1E and S1B). The phospho-specific antibody was unable to recognize S13A mutant NF2 (Figs. 1D and S1C). We also aligned amino acid sequences of different NF2 orthologs, and found that S13 was conserved in vertebrates, suggesting that a vertebrate-specific regulatory mechanism emerged late in evolution (Fig. 1F). These results indicate that S13 is a novel site on NF2 subjected to phosphorylation-dependent regulation.

**Figure 1.**
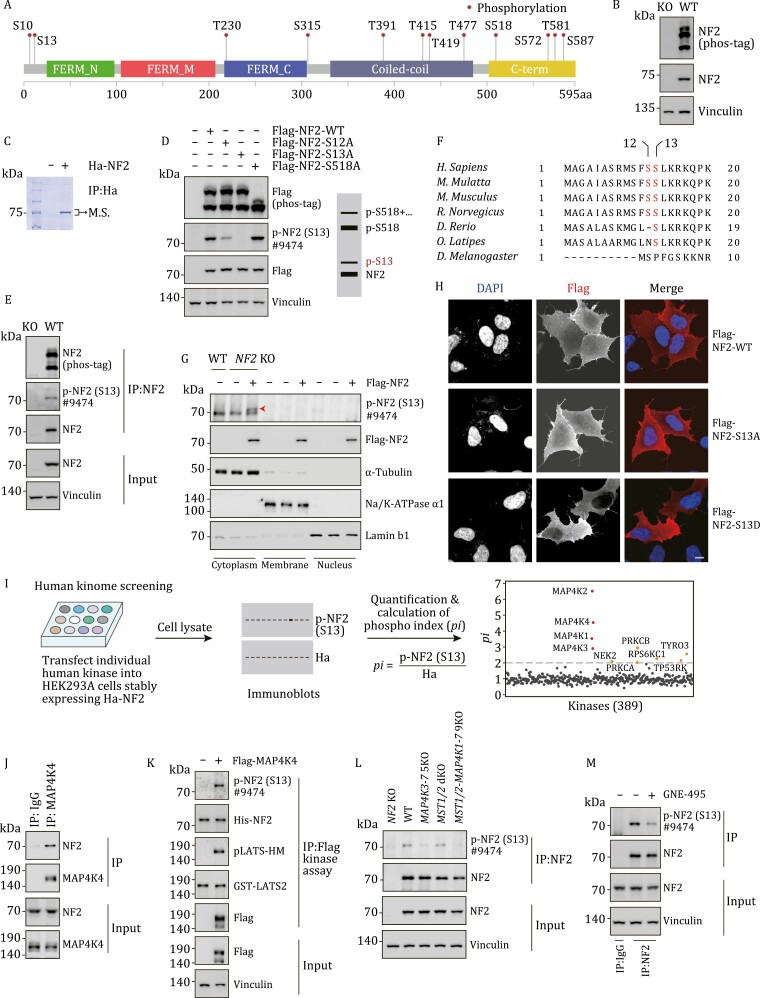
NF2 is phosphorylated at S13 by MAP4K family kinases. (A) Phosphorylation of NF2. Information on phosphorylated sites was from PhosphoSite. FERM, coiled-coil and C-term domains were indicated. (B) NF2 is phosphorylated at multiple sites. Endogenous NF2 was separated into multiple bands on a phos-tag gel. Antibody specificity was reflected by the lack of signals in *NF2* KO cells. (C) Purification of NF2 for mass spectrometry analysis. Ha-tagged NF2 was expressed in HEK293A cells, and proteins precipitated by Ha antibody conjugated-beads were separated by SDS-PAGE. The band corresponding to NF2 was cut and subjected to mass spectrometry. (D) Phosphorylation at S13 and S518 exhibits different mobilities. WT and mutated NF2 were separated into four bands on phos-tag gels, and positions of S13 and S518 phosphorylated NF2 were indicated. (E) Validation of NF2 S13 phospho-specific antibody. Antibody #9474 effectively detected immunoprecipitated NF2. (F) S13 is conserved in vertebrate NF2. Amino acid sequences of NF2 orthologs were aligned, and S12 and S13 were highlighted in red. (G) S13 phosphorylated NF2 is mainly present in cytoplasmic fraction. Cell lysate was fractionated into nucleus, cytoplasm, and membrane. The amount of NF2 protein in Flag and p-NF2 (S13) blots was normalized. Arrow head indicates S13 phosphorylated NF2. (H) S13D NF2 shows compromised plasma membrane localization. HEK293A cells transfected with WT, S13A, or S13D NF2 were subjected to immunofluorescence using Flag antibody. Scale bar, 10 μm. (I) MAP4K family kinases phosphorylate NF2 at S13. Human kinome screen: Human serine and threonine kinases were expressed in HEK293A cells stably expressing Ha-NF2. Phospho-S13 signal and total NF2 signal were obtained to determine phospho index (*pi*). (J) NF2 interacts with MAP4K4. Endogenous coimmunoprecipitation assay was performed. (K) MAP4K4 phosphorylates NF2 *in vitro*. Bacterially expressed His-NF2 was purified and used as kinase substrate. Flag-MAP4K4 was expressed, immunoprecipitated, and used for kinase assay. The phosphorylation of GST-LATS2 by MAP4K4 was used as a control. (L) Knockout of *MAP4K3–7* blocks NF2 phosphorylation at S13. MAP4K1/2 were not significantly expressed in HEK293A cells. *MST1*/*2* KO showed no significant effect on NF2 phosphorylation. (M) MAP4K inhibitor represses NF2 phosphorylation at S13. GNE-495 is a MAP4K4 inhibitor that also targets other MAP4Ks.

NF2 localizes at different subcellular domains, including the plasma membrane (McClatchey and Giovannini 2005). The amino (N) terminus (aa 1–18) of NF2 contains four serine residues, and the phosphorylation of these serine residues is critical in regulating NF2 localization at the plasma membrane (Fig. 1F) (Cole et al., 2008). Indeed, as shown by immunofluorescence staining, wild-type NF2 and 4SA non-phosphorylated mutant, in which four serine residues were replaced by alanine, showed distinct localization at membrane and cell–cell boundaries. On the other hand, NF2 lacking N-terminal residues (Δ1–18) and 4SD phospho-mimic mutant, in which four serine residues were replaced by aspartic acid, were not enriched at plasma membranes (Fig. S2A). These results are consistent with previous findings (Cole et al., 2008), and indicate a critical role of NF2 phosphorylation at N-terminal residues in regulating its subcellular localization.

Among the four serine residues at the N-terminal motif of NF2, only S13 was effectively phosphorylated at the target band (Fig. S2B). We then determined if the phosphorylation of S13 is involved in regulating the subcellular localization of NF2. Total cell lysates were fractionated into nuclear, cytoplasmic, and membrane fractions, and the amount of NF2 protein was normalized. Interestingly, we found that only NF2 in the cytoplasmic fraction was significantly phosphorylated at S13 (Fig. 1G). In agreement with the fractionation data, plasma membrane localization of the S13D mutant mimicking S13 phosphorylation, but not the non-phosphorylated S13A mutant, was significantly reduced (Fig. 1H), suggesting that S13 phosphorylation acts as a switch for NF2 subcellular localization, either discharging NF2 from the plasma membrane or inhibiting NF2 membrane targeting.

We next interrogated if phosphorylation of NF2 at S13 affects Hippo signaling components. YAP and TAZ phosphorylation were dramatically reduced in *NF2* knockout (KO) HEK293A cells, and rescued by wild type (WT) NF2 (Fig. S3A). However, phospho-null (S13A) or phospho-mimic (S13D) NF2 mutants effectively induced YAP and TAZ phosphorylation in *NF2* KO cells and reduced the expression of YAP/TAZ target genes (Fig. S3A and S3B). In contrast, several cancer-derived mutations, including L46R, F62S, L64P, and L141P, which were located in FERM domains and might render NF2 defective in LATS1/2 binding, failed to effectively induce YAP and TAZ phosphorylation (Fig. S3C). These results suggest that, when expressed at similar levels, S13A or S13D mutant NF2 regulates YAP and TAZ phosphorylation in a manner comparable to WT NF2.

To assess the role of NF2 S13 phosphorylation *in vivo*, we established knockin mice with S13 mutated into alanine (S13A) or aspartic acid (S13D) to mimic non-phosphorylated or phosphorylated S13, respectively (Fig. S4A–C). We prepared mouse embryonic fibroblasts (MEF) from WT, S13A, or S13D mice, and analyzed their effects on Hippo signaling. YAP and TAZ phosphorylation were not significantly changed in cells with one or both *Nf2* alleles replaced by S13A or S13D (Fig. S4D). It is known that NF2 regulates protein stability of motin family proteins including angiomotin (AMOT), AMOTL1, and AMOTL2 (Wang et al., 2021). However, the protein levels of AMOT, AMOTL1, and AMOTL2 were not changed in S13A or S13D MEF cells (Fig. S4E). Together, these data indicate that S13 phosphorylation does not directly regulate the Hippo pathway.

To identify the kinase(s) responsible for NF2 S13 phosphorylation, we performed a human kinome screen by establishing a cell line stably expressing Ha-NF2, then transiently expressing kinases individually and monitoring their effects on NF2 S13 phosphorylation by immunoblotting (Fig. 1I). In this human kinome screen, the most notable kinases that effectively induced S13 phosphorylation were the MAP4K family kinases (MAP4K1–7, MAP4Ks), most of which showed similar effects on NF2 S13 phosphorylation (Figs. 1I and S5A; Table S1). MAP4K4 was used hereafter for mechanistic studies (Fig. S5B). Several other kinases, including protein kinase C (PKC) family proteins PRKCA and PRKCB, also showed activity toward NF2 S13 phosphorylation, although their effects were much weaker (Fig. 1I). We treated cells with Go6983, a pan PKC inhibitor, and observed no significant effect on NF2 S13 phosphorylation, while the phosphorylation of PKC substrates MARCKS were dramatically reduced (Fig. S5C). The potential effect of PKC was not followed further in this study.

The effect of MAP4K4 on NF2 S13 phosphorylation was specific, in a manner independent on S518 (Fig. S5D). MAP4K4 physically interacted with NF2, as demonstrated by reciprocal co-immunoprecipitation assays (Figs. 1J and S5E). In an *in vitro* kinase assay, MAP4K4 directly phosphorylated NF2 at S13, similar to its effect on LATS2, a known MAP4K4 substrate (Fig. 1K) (Meng et al., 2015, Zheng et al., 2015). However, in cells with *MAP4K4* deletion, the phosphorylation of NF2 was not decreased, indicating potential redundancy among MAP4Ks (Fig. S5F). To further validate the effects of MAP4K1–7 on NF2 phosphorylation, we utilized a cell line deficient in MAP4K3–7 (*MAP4K3–7* 5KO) (Meng et al., 2015), and observed significantly reduced S13 phosphorylation (Fig. 1L). Moreover, NF2 S13 phosphorylation was drastically reduced in cells treated with GNE-495, a small molecule inhibitor for MAP4Ks (Fig. 1M) (Vitorino et al., 2015). On the other hand, NF2 S13 phosphorylation was intact in cells lacking MST1/2, despite the two kinases sharing high homology with MAP4K1–7 (Fig. 1L). We also studied the effect of MAP4Ks on the subcellular localization of NF2. In a fractionation assay, cells with ectopic MAP4K4 expression showed increased cytoplasmic NF2 (Fig. S5G). Conversely, in *MAP4K3–7* 5KO cells, the plasma membrane staining of endogenous NF2 was enriched (Fig. S5H). These gain- and loss-of-function studies collectively demonstrate that S13 phosphorylation and subcellular localization of NF2 are directly regulated by MAP4Ks.

MAP4K4 is required for developmental and pathological angiogenesis (Vitorino et al., 2015). Although moesin has been proposed as a mediator of MAP4K4 function in angiogenesis (Vitorino et al., 2015), additional modulators may exist. Since S13 phosphorylation by MAP4Ks regulates NF2 localization at the plasma membrane and cell-cell junctions, it is possible that this phosphorylation event is involved in MAP4K4-regulated angiogenesis.

We then tested if NF2 S13 phosphorylation by MAP4Ks played a role in endothelial cells. In human umbilical vein endothelial cells (HUVEC), the phosphorylation of NF2 S13 was gradually decreased following MAP4K inhibitor (GNE-495) treatment, and induced upon MAP4K4 overexpression (Fig. S6A and S6B). We also isolated and cultured liver endothelial cells from WT, S13A, or S13D mice, and observed a significant reduction of plasma membrane localization of NF2 S13D (Fig. S6C). These results indicate that the regulation of NF2 S13 phosphorylation by MAP4Ks is conserved in endothelial cells.

To determine a potential role of NF2 and S13 phosphorylation on angiogenesis, we performed tube formation assays using HUVEC cells. We observed that the tube formation abilities, as measured by the total length of tubes, the number of meshes, and the number of junctions, were dramatically reduced in NF2 knockdown cells, whereas increased in NF2 overexpressing cells (Fig. S6D and S6E). However, the ability of WT, S13A, or S13D NF2 in promoting capillary formation was largely indistinguishable (Fig. S6E). Thus, NF2 is indispensable for capillary formation *in vitro*, but the effect of S13 phosphorylation is not robust.

The intraretinal vasculature of mice is immature at birth and keeps developing postnatally in a tightly regulated manner (Stahl et al., 2010). Therefore, we analyzed the effects of S13A and S13D mutations on angiogenesis during postnatal retinal development. There was no difference in body weight of all mice during the assay (Fig. S7A). Seven-day-old retina was harvested and the vasculature was visualized by staining with isolectin B4 (IB4). Compared with WT mice, both S13A and S13D knockin mice showed normal retinal angiogenesis, as assessed by the endothelial cell-covered areas, number of sprouts per angiogenic front, average sprout length, vascular outgrowth, number of branch points, and tip cell morphology (Fig. S7B–H). We also compared superficial and deep vessels of retinas with different genotypes, and found no significant differences between S13A/D and controls (Fig. S7I). Together, these results suggest that the phosphorylation of NF2 at S13 is dispensable during postnatal retinal angiogenesis.

MAP4K4 has been shown to be involved in pathological angiogenesis (Vitorino et al., 2015). Thus, we determined whether NF2 S13 phosphorylation plays a role in pathological angiogenesis. Developmental disorders and premature birth are often associated with altered retinal vasculature. For instance, preterm infants are frequently exposed to high oxygen, which may cause obliteration of immature vessels, and in some cases, the vascular injury cannot be fully repaired and undergoes pathological neovascularization (NV), and this oxygen-induced retinopathy (OIR) has been faithfully modeled in mice (Stahl et al., 2010).

In OIR mouse model, seven-day-old mice are subjected to 75% oxygen for 5 days, followed by normal room air for another 5 days. The OIR retina usually undergoes a vaso-obliteration phase under hyperoxia and a neo-angiogenesis phase under normoxia, and cell clusters (tufts) are often observed during neo-angiogenesis (Fig. 2A). WT, S13A, and S13D mice were subjected to OIR, and all mice used in this experiment had similar body weight (Fig. 2B). However, OIR retina of S13A mice exhibited significantly larger avascular area and smaller sprouting areas than control and S13D retina (Fig. 2C–E). On the other hand, the neovascular tufts of these mice were similar (Fig. 2C and 2F). These results indicate that S13 phosphorylation of NF2 is critical for endothelial cell sprouting during retinal vessel repair following hyperoxia.

**Figure 2.**
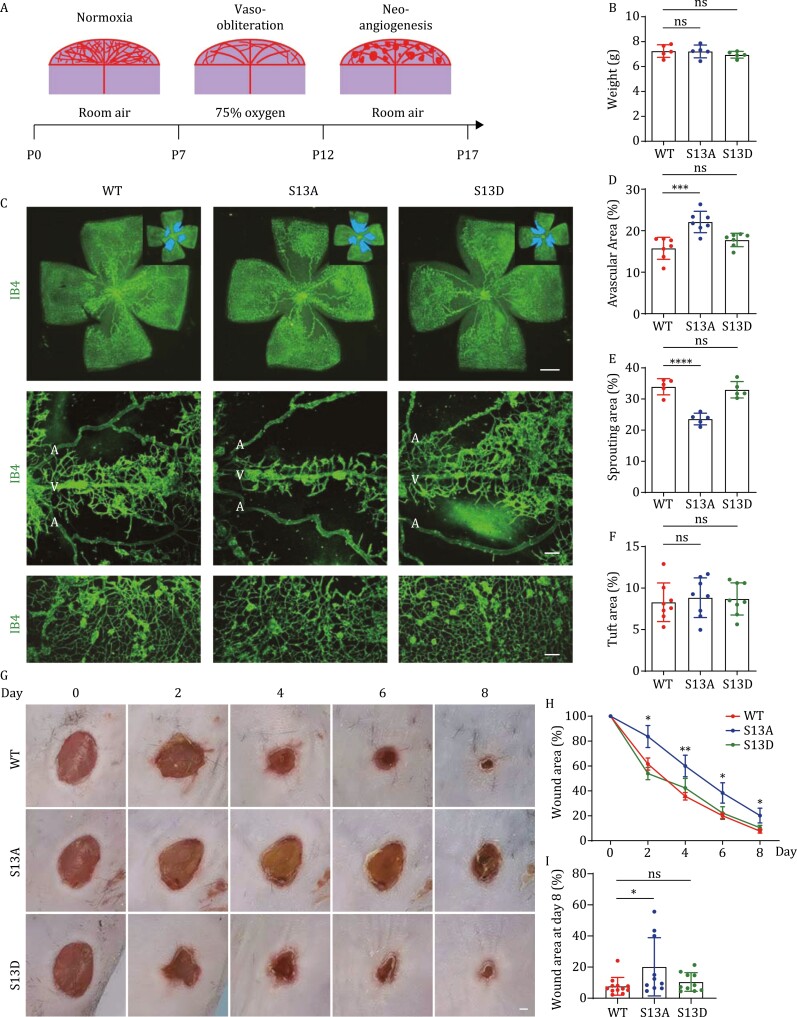
NF2 S13 phosphorylation promotes pathological angiogenesis. (A) Model of oxygen-induced retinopathy on mouse pups. Pups at P7 were subjected to 75% oxygen for 5 days, followed by normal room air for another 5 days. (B) Quantification of body weight of pups at P17 after OIR. (C) IB4-stained whole-mount retinal vasculature (scale bar, 1 mm), vessel sprouting from veins (scale bar, 100 μm), and neovessel tufts (scale bar, 100 μm) of P17 mice after OIR. Compared with control and S13D mice, S13A mice showed smaller vascular and sprouting areas. White colored letter A and V indicate artery and vein, respectively. The neovascular tufts were similar in WT, S13A and S13D retina. (D) Quantification of avascular area in the whole retinas. (E) Quantification of sprouting area from veins. (F) Quantification of tuft area after OIR. (G) Representative images of wound healing of mice from day 0 to day 8 after cutaneous punch biopsy. The wound healing process of S13A mice was significantly retarded. Scale bar, 1 mm. (H) Quantification of percentage of wound area after wounding. (I) Quantification of wound area at day 8 after wounding.

Angiogenesis plays a crucial role in wound healing. During the early phase of wound healing, angiogenic capillaries invade the wound clot and organize into a microvascular network throughout the new stroma (Potente et al., 2011). We performed wound healing assay on WT, S13A, and S13D mice by generating identical wounds in each mouse after cutaneous punch biopsy and then monitoring wound closure for 8 days (Fig. 2G). Compared to WT and S13D mice, the wound healing process in S13A mice was significantly retarded (Fig. 2H and 2I), suggesting that NF2 S13 phosphorylation is critical for cutaneous wound healing. It is noteworthy that S13A/D mutation is not restricted in endothelial cells, in addition to angiogenesis, other cell types may also contribute to the phenotypes observed in wound healing process.

In summary, this study revealed that S13 of NF2 is phosphorylated by MAP4K family kinases (MAP4K1–7), and this post-translational modification is involved in pathological angiogenesis. Recently, a study has shown that TNIK (MAP4K7) phosphorylates NF2 at S13 and promotes tumorigenesis in lung squamous cell carcinoma (LSCC) (Torres-Ayuso et al., 2021). Our results indicate that, in addition to MAP4K7, 6 other MAP4K family kinases (especially MAP4K2/4) are bona fide kinases responsible for NF2 S13 phosphorylation. The phosphorylation of NF2 at S13 is critical in pathological angiogenesis, but dispensable in developmental angiogenesis (Figs. 2 and S5). We reasoned that the activity of MAP4Ks during developmental angiogenesis might be low and the effect of NF2 S13 phosphorylation could be compensated by other mechanisms, whereas the activity of MAP4Ks during pathological angiogenesis might be high, and upregulation of NF2 S13 phosphorylation might play a more dominant role. In support of this, it has been shown that in OIR mice, TLR4-mediated signaling can activate MAP4K4 and promote angiogenesis (Chen et al., 2019). S13 phosphorylation and cytoplasmic localization of NF2 may be required for remodeling of endothelial cells, and in S13A mutant cells, this remodeling process may be blocked to prevent rapid vascular sprouting.

By using knockin mouse models, this study demonstrated an unequivocal role of NF2 S13 phosphorylation in pathological angiogenesis. However, whether NF2 S13 phosphorylation mediates the effect of MAP4K family proteins in angiogenesis remains unclear and requires further epistatic analysis combining *Nf2* S13 mutants with *Map4k1–7* deletions. Nevertheless, the involvement of NF2 S13 phosphorylation in pathological angiogenesis indicates that activators or inhibitors of MAP4Ks could be used to treat diseases associated with aberrant angiogenesis.

## Supplementary Material

pwac005_suppl_Supplementary_Material

pwac005_suppl_Supplementary_Table

## Footnotes

This study is supported by grants from the National Key R&D program of China (2018YFA0800304, 2020YFA0803202), the National Natural Science Foundation of China (81772965), and Science and Technology Commission of Shanghai Municipality (19JC1411100, 21S11905000) to FXY.

All authors declare no potential conflict of interest.

All mice were maintained under specific pathogen-free conditions and handled according to the Animal Ethics Committee of Shanghai Medical College, Fudan University.

Conceptualization: MM and FXY; methodology: MM, ZZ, YW, YZ, YG, RJ, ZM, and FXY; formal analysis: MM and FXY; investigation: MM, ZZ, YW, YZ, YG, RJ, ZM, and FXY; resources: ZM and FXY; writing of the original draft: MM and FXY; review and editing of the manuscript: MM, ZM, YW and FXY; supervision: FXY; and funding acquisition: FXY.
