# Supplementary material for: Phosphorylation of NF2 at Serine-13 by MAP4K family kinases mediates pathological angiogenesis
Source: Protein Cell. 2022 Oct 14;14(2):137–42. doi: 10.1093/procel/pwac005 (PMC10019571; doi:10.1093/procel/pwac005)
Supplement: pwac005_suppl_Supplementary_Material [file pwac005_suppl_supplementary_material.pdf]

Supplemental materials

Materials and methods

Mice

All mice were maintained under specific pathogen-free conditions and handled according to the Animal Ethics Committee of Shanghai Medical College, Fudan University. *Nf2* S13A and S13D knockin mice were generated in Shanghai Model Organisms Center. The genotypes of mice were determined by PCR.

Cell culture

HEK293A cells were maintained in DMEM medium supplemented with 10% fetal bovine serum (FBS, Gibco) and 50 μg/mL penicillin/streptomycin at 37 ℃ in the incubator containing 5% CO2. *MAP4K3-7* 5KO, *MST1/2* dKO, and *MST1/2-MAP4K1-7* 9KO cells were described previously (Meng *et al*, 2015). *NF2* KO and *MAP4K4* KO cells were generated by CRISPR-Cas9 system. Mouse embryonic fibroblasts (MEF) cells were maintained in DMEM medium containing 10% FBS and 50 μg/mL penicillin/streptomycin. Human umbilical vein endothelial cells (HUVEC) were cultured in Endothelial Cell Growth Medium 2 (PromoCell, C-22011) on plates pre-coated with 1% collagen (Corning, 354236).

Plasmids

For transient overexpression, genes were cloned into pLVX-puro vector (Takara, #632164) with Flag-, Ha- or Myc-tag. Transfection procedures follow the instructions of PolyJet™ DNA *In Vitro* Transfection Reagent (Signagen, #SL100688). For lentiviral infection, plasmids with pLVX vector were co-transfected with PsPAX2 and pMD.2g in HEK293A cells. After 48 h, lentivirus containing medium was filtered through a 0.45-μm filter, then used for cell infection with 5 mg/mL polybrene. One day after infection, cells were replaced with fresh culture medium containing puromycin (Invivogen).

For gene deletion, CRISPR-Cas9 genomic editing technology was used in this study. The plasmid px459 v2 was provided by Dr. Feng Zhang (Addgene #62988). The single-guide RNA (sgRNA) sequences targeting individual genes were as follows: *NF2* #1, GTCCATGGTGACGATCCTCA; *NF2* #2, CGTCACCATGGACGCCGAGA; *NF2* #3, GATCCTCACGGTGAACGTCT; *MAP4K4* #1, CAGGACATGATGACCAACTC; *MAP4K4* #2, GGGCGGAGAAATACGTTCAT.

Kinome screening

HEK293A stable cells expressing Ha-tagged NF2 were constructed. Flag-tagged human kinome plasmids (Meng *et al.*, 2015) were transfected into the stable cells individually. Samples were lysed in SDS loading buffer and subjected to immunoblotting. Phospho-specific antibodies for p-NF2 (S13) were used to detect signals.

Kinase assay

Full-length His-tagged human NF2 protein was purified from *Escherichia coli* (BL-21). GST-tagged mouse LATS2 protein was expressed in *MST1/2-MAP4K1-7* 9KO HEK293A cells. After immunoprecipitation from transfected HEK293A cells, MAP4K4 was captured on the beads and incubated with the substrates His-NF2 or GST-LATS2. The kinase reaction was performed for 30 min at 30 ℃. Phospho-specific antibodies for p-NF2 (S13) and p-LATS (T1079) were used to evaluate the kinase activity of MAP4K4.

Immunoblotting

Cells were lysed in SDS loading buffer containing 50 mM Tris pH 6.8, 2% SDS, 0.025% bromophenol blue, 10% glycerol, and 5% BME. Protein lysates were separated by sodium dodecyl sulfate-polyacrylamide gel electrophoresis (SDS-PAGE) and transferred onto nitrocellulose membrane. 7.5% phos-tag gel was used to resolve the phospho-NF2 and phospho-YAP proteins. The membrane was blocked in 5%

non-fat milk for 1 h at room temperature, and incubated with primary antibodies in 5% bovine serum albumin (BSA) overnight at 4 ℃. The membrane was then incubated with secondary HRP-conjugated antibodies in 5% non-fat milk for 1 h. Molecular weight marker, ECL substrates, and image acquisition equipment (5200S) were from Tanon Science & Technology Co., Ltd. p-NF2 (S13) rabbit polyclonal antibody was custom-made by Abclonal.

Immunoprecipitation

Cells were lysed in mild lysis buffer (50 mM Tris-HCl at pH 7.5, 150 mM NaCl, 1% NP-40, 50 mM NaF) supplemented with protease inhibitor and phosphatase inhibitor cocktails and 1 mM PMSF. Cell lysates were then incubated with antibodies for 1 h at 4 ℃, and with protein A agarose beads (Repligen, 10-1003-02) for another 1 h. Beads were washed 4 times with mild lysis buffer. Proteins were captured on the beads, dissolved in SDS loading buffer, and subjected to immunoblotting. Antibodies used were as follows: NF2 (Cell Signaling Technology, 12888), Flag-Agarose (Smart-Lifesciences, SA042C), and Ha-tag (BIOT Biology, 0101006).

Immunofluorescence

Cells were seeded on fibronectin-coated coverslips and incubated with medium. Cells were then fixed in 4% paraformaldehyde for 15 min, treated with 0.1% Triton X-100 for 10 min, and blocked with 3% goat serum for 1 h. Cells were incubated with primary antibodies overnight at 4 ℃ and Alexa Fluor 488- or Alexa Fluor 594-conjugated secondary antibodies for 1 h at room temperature. Slides were mounted with ProLong™ Gold Antifade Mountant with DAPI (ThermoFisher, P36935). After mounting, images were captured with Leica SP8 or Zeiss confocal microscope.

RNA extraction, reverse transcription, and real-time PCR

Total RNA was extracted using the RNeasy Plus mini kit (Qiagen). cDNA was generated using the PrimeScript RT reagent Kit (TaKaRa), and quantitative qPCR was conducted using SYBR Green qPCR Master Mix (TaKaRa) on the 7500 real-time

PCR system (Applied Biosystems). Relative abundance of mRNA was calculated by normalization to β-actin mRNA. Primers used in PCR were as follows: *CYR61*, F:5'-AGCCTCGCATCCTATACAACC-3', R:5'-TTCTTTCACAAGGCGGCACTC-3'; *CTGF*, F:5'-CCAATGACAACGCCTCCTG-3', R:5'-TGGTGCAGCCAGAAAGCTC-3'; *β-ACTIN*, F:5'-GCCGACAGGATGCAGAAGGAGATCA-3', R:5'-AAGCATTTGCGGTGGACGATGGA-3'.

Isolation of mouse embryonic fibroblasts (MEF)

MEF cells were isolated from mouse embryonic tissue at E11-13. The head above eyes and red tissue (heart and liver) of embryo were cut off. The rest of the embryo were dissected, minced into fragments, and then digested with collagenase/dispase (Roche, 1 mg/mL) in DMEM at 37°C for 30 min and filtered through 40 μm nylon mesh. After centrifugation, the supernatant was removed. MEF cells were resuspended in fresh medium and seeded on the dish.

Isolation of mouse liver endothelial cells

Endothelial cells were isolated from mouse liver between 6 and 8 weeks. The liver was minced, digested with collagenase/dispase (Roche, 1 mg/mL), and dispersed into a single cell suspension. The endothelial cells were purified from cell suspension with CD31 MicroBeads (Miltenyi Biotec, 130-097-418) using a strong magnetic separation method. The purified cells were cultured on dishes pre-coated with 1% collagen in Endothelial Cell Growth Medium 2.

Tube formation assay

HUVECs were used in the tube formation assay. For gene knockdown, siRNA was transfected into HUVECs by Lipofectamine™ RNAiMAX Transfection Reagent (ThermoFisher, 13778075). The sequences of siRNA were as follows: si*NF2*, GGACAAGAAGGTACTGGATCATGAT. For tube formation assay, HUVECs were resuspended in medium with a concentration of $3x10^5$/mL. Then 200 μL cell

suspension were seeded in 48-well plates pre-coated with growth factor reduced Matrigel (Corning, 356231), and underwent angiogenesis *in vitro* for 8 h. Images were recorded by Olympus microscope. The total length, junctions and meshes of tubes were analyzed by ImageJ.

Whole-mount staining of retinas

The eyes of mouse pups were fixed in 4% paraformaldehyde (Sigma) for 1 h on ice. After dissection, retinas were blocked in 0.5% blocking reagent (PerkinElmer, FP1012) with TNT buffer (150 mM NaCl, 100 mM Tris-HCl at pH 7.5 and 0.4% Triton X-100) for 2 h at room temperature, and then incubated with Alexa-Fluor-conjugated isolectin B4 (Invitrogen, I21411) overnight at 4 ℃. The retinas were then flat-mounted with Fluromount-G (Invitrogen) and examined using Leica SP8 or Zeiss confocal microscope.

Oxygen-induced retinopathy (OIR)

The protocols of OIR were described previously (Smith *et al*, 1994). In the pathologic mouse model, breeding mother and P7 neonatal pups were exposed to 75% O_2 until P12. Then, pups were exposed to room air for 5 days until P17. Eyes were collected and the retinas were stained with Alexa-Fluor-conjugated isolectin B4. The avascular, sprouting, and tuft areas were measured by ImageJ.

Wound healing assay

In vivo wound healing assay was described previously (Zhang *et al*, 2016). Mice aged between 6-8 weeks were anesthetized and shaved on the back. Wounds were created using a 6-mm biopsy punch on the back skin. This assay set two wounds per mouse, one on each side of the dorsal midline, at equal distance, so that the tension of skin would be equal all over the mouse back. The images were acquired with digital camera from day 0 to day 8. Wound areas were measured by ImageJ.

Statistical analysis

All experiments subjected to statistical test were repeated at least three times. Statistical analyses were performed using GraphPad Prism8. The independent experimental data were used for Student's t-test. $P < 0.05$ was considered statistically significant (*$P < 0.05$; **$P < 0.01$; ***$P < 0.001$).

Supplemental References

Meng Z, Moroishi T, Mottier-Pavie V, Plouffe SW, Hansen CG, Hong AW, Park HW, Mo JS, Lu W, Lu S *et al* (2015) MAP4K family kinases act in parallel to MST1/2 to activate LATS1/2 in the Hippo pathway. *Nat Commun* 6: 8357

Smith LE, Wesolowski E, McLellan A, Kostyk SK, D'Amato R, Sullivan R, D'Amore PA (1994) Oxygen-induced retinopathy in the mouse. *Invest Ophthalmol Vis Sci* 35: 101-111

Zhang F, Prahst C, Mathivet T, Pibouin-Fragner L, Zhang J, Genet G, Tong R, Dubrac A, Eichmann A (2016) The Robo4 cytoplasmic domain is dispensable for vascular permeability and neovascularization. *Nat Commun* 7: 13517

Supplementary Figure Legends

Figure S1

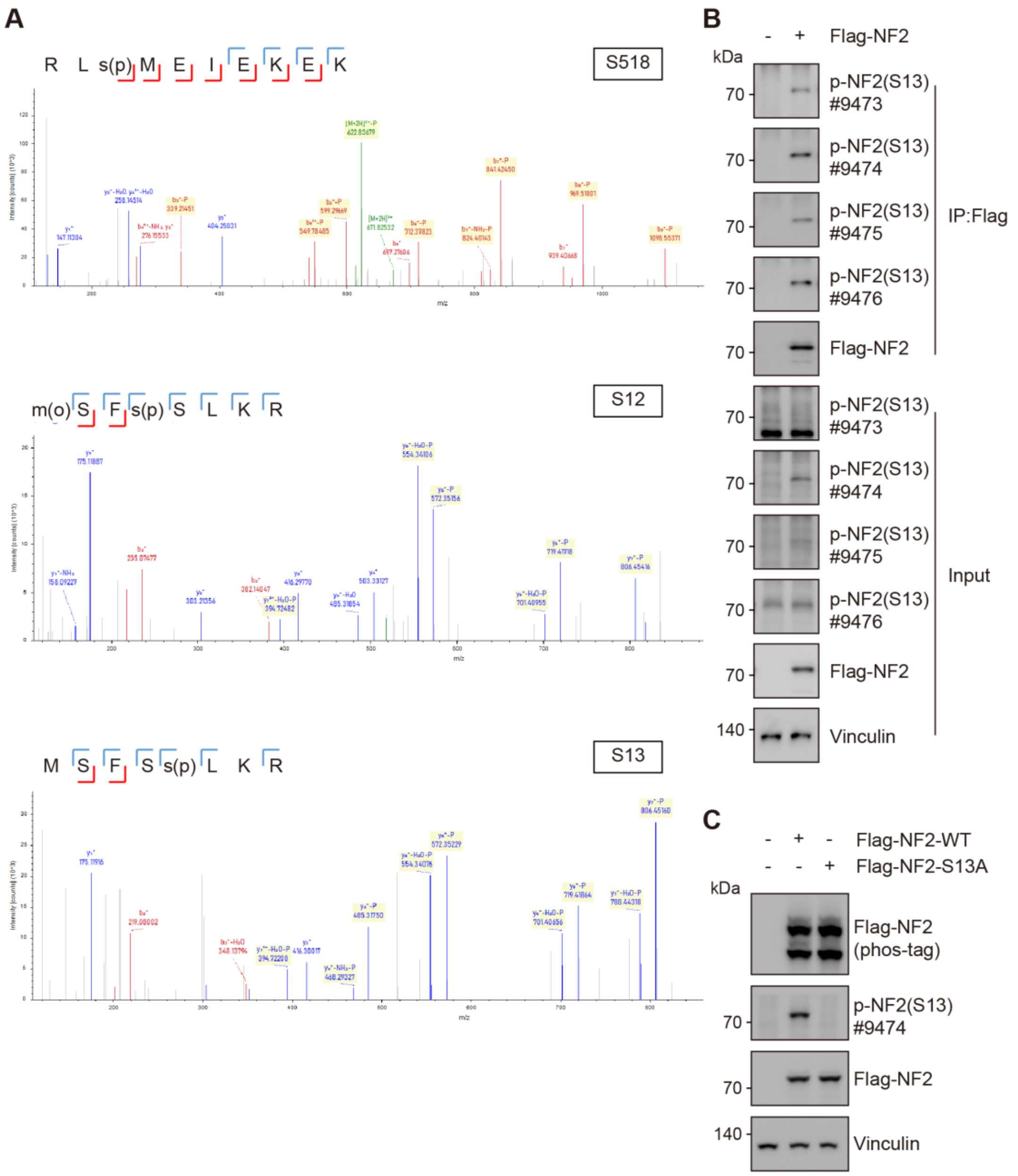

Figure S1. Phosphorylation of NF2 at S13.

(A) Phosphorylation at S12, S13, and S518 of NF2 as shown by mass spectrometry.

(B) Validation of different pS13 antibodies. HEK239A cells expressing Flag-NF2 were used. Whole cell lysate or precipitated proteins (by Flag antibody) were used to test affinity and specificity of pS13 antibodies.

(C) Specificity of pS13 antibody. NF2 WT and S13A mutant were transfected into HEK293A cells. pS13 antibody failed to recognize S13A mutant.

Figure S2

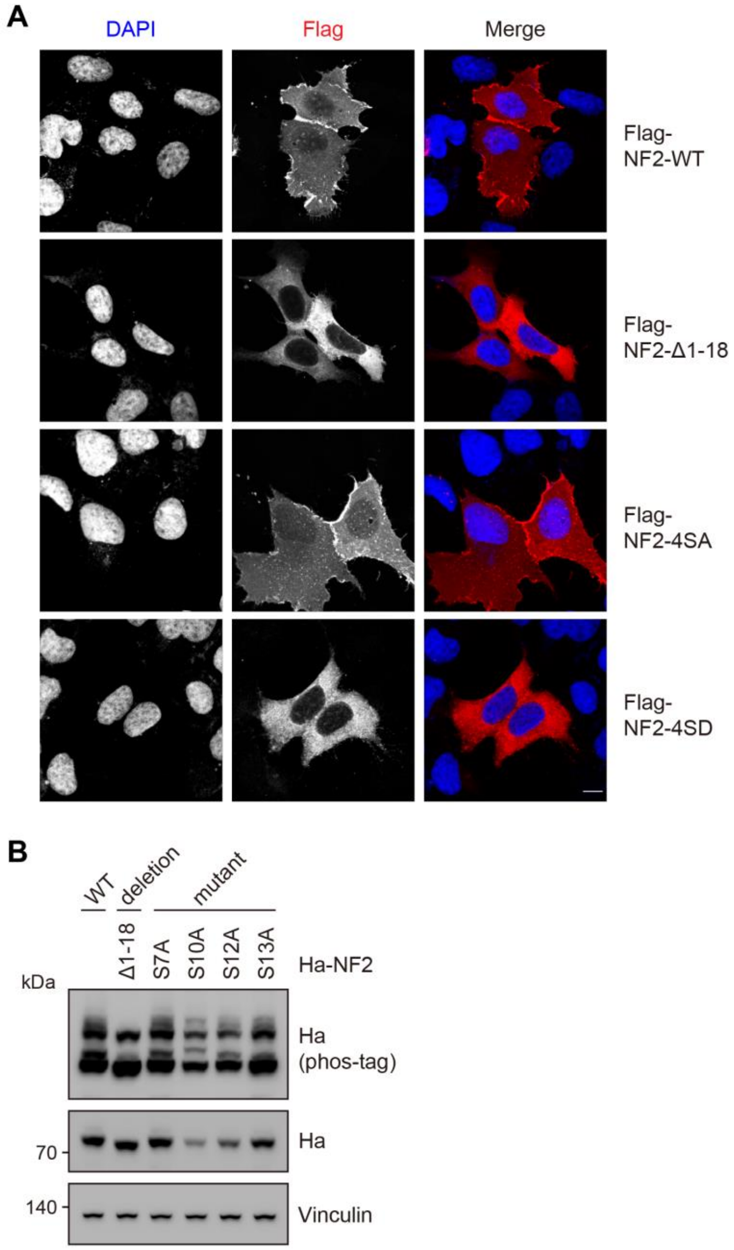

Figure S2. The effect of NF2 N-terminal residues.

(A) Subcellular localization of WT and mutated NF2. WT or mutated NF2 was transfected into HEK293A cells, and subcellular localization was determined by immunofluorescence staining. Scale bar, 10 μm.

(B) Four serine residue mutants separate on the phos-tag gel. S13A but not other mutants showed compromised phosphorylation.

Figure S3

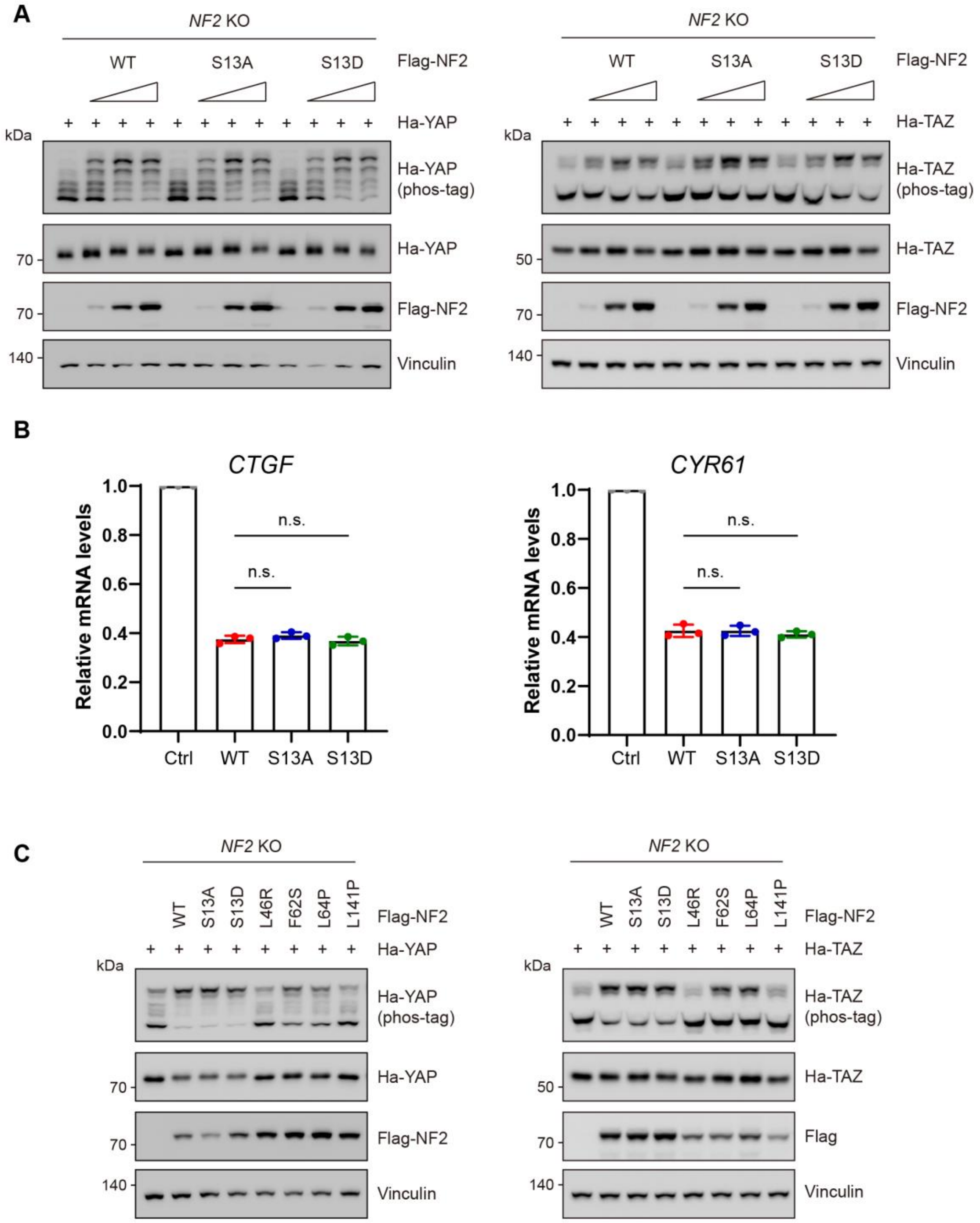

Figure S3. NF2 S13 phosphorylation does not regulate YAP/TAZ activity.

(A) Effects of NF2 WT, S13A, and S13D on YAP/TAZ phosphorylation. NF2 WT and mutants induced YAP/TAZ phosphorylation in HEK293A *NF2* KO cells.

(B) Effects of NF2 WT, S13A, and S13D on the expression of YAP/TAZ target genes. The mRNA level of *CYR61* and *CTGF* was effectively downregulated in *NF2* KO HEK293A cells expressing WT, S13A, or S13D NF2.

(C) Several cancer-derived NF2 mutants are less effective in inducing YAP/TAZ phosphorylation. NF2 L46R, F62S, L64P, and L141P mutant, but not S13A or S13D, when transfected into *NF2* KO HEK293A cells, showed compromised ability to induce YAP/TAZ phosphorylation.

Figure S4

A

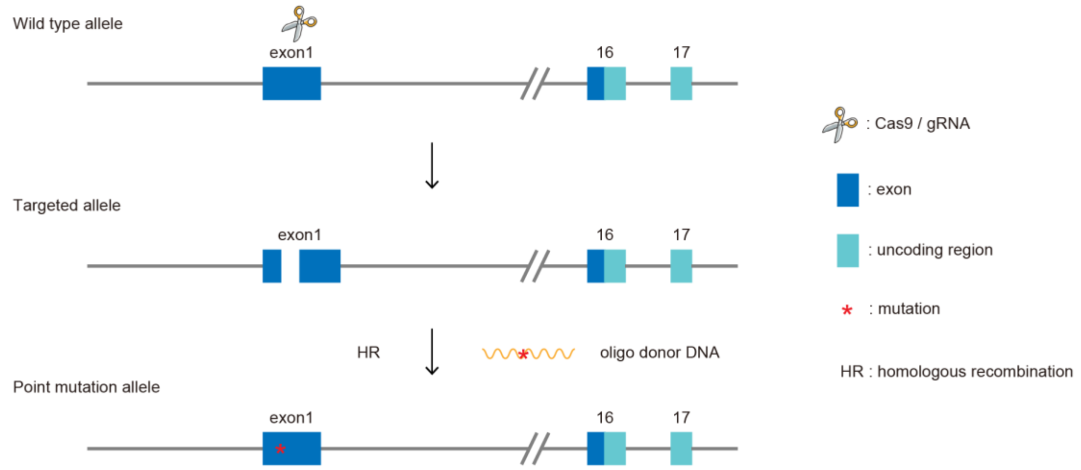

B

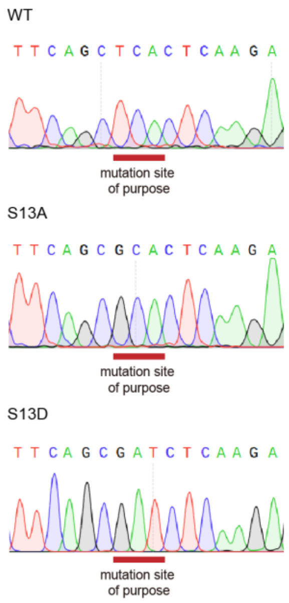

D

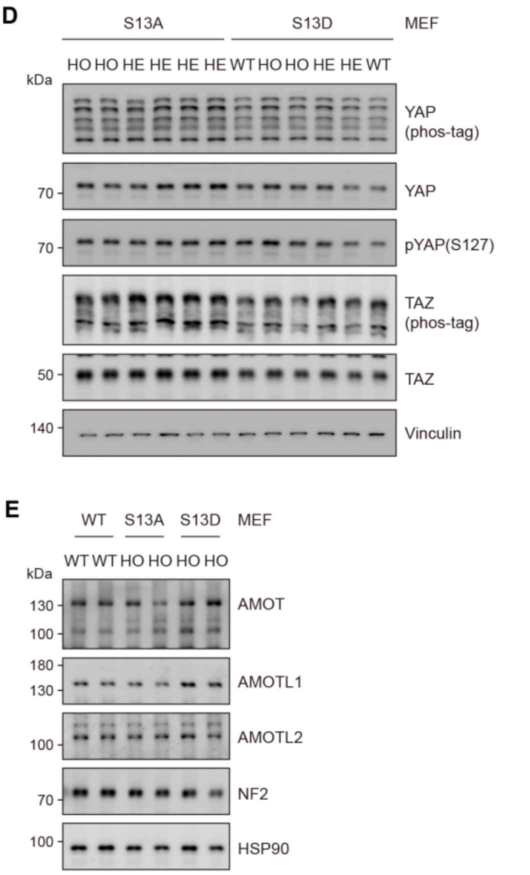

C

Primer	Sequence (5' → 3')
Forward	AGGAGGCGGGACGAAGCGGAAGT
Reverse	CGGGAAATGCCAAAACCAAGAACC

Figure S4. Establishment of *Nf2* S13A and S13D knockin mice.

(A) Targeting strategy: The first exon was targeted in *Nf2* S13A and S13D knockin mouse model, facilitated by CRISPR/Cas9 technology.

(B) Sequencing results of knockin mice.

(C) PCR primers used for genotyping.

(D) Effects of NF2 WT, S13A, and S13D on YAP/TAZ phosphorylation in MEF. There was no significant change between *Nf2* WT and mutant MEF cells.

(E) The expression of angiomotin family proteins is not regulated by S13 phosphorylation. The protein levels of AMOT, AMOTL1, and AMOTL2 were not changed in WT, S13A, and S13D MEF cells.

Figure S5

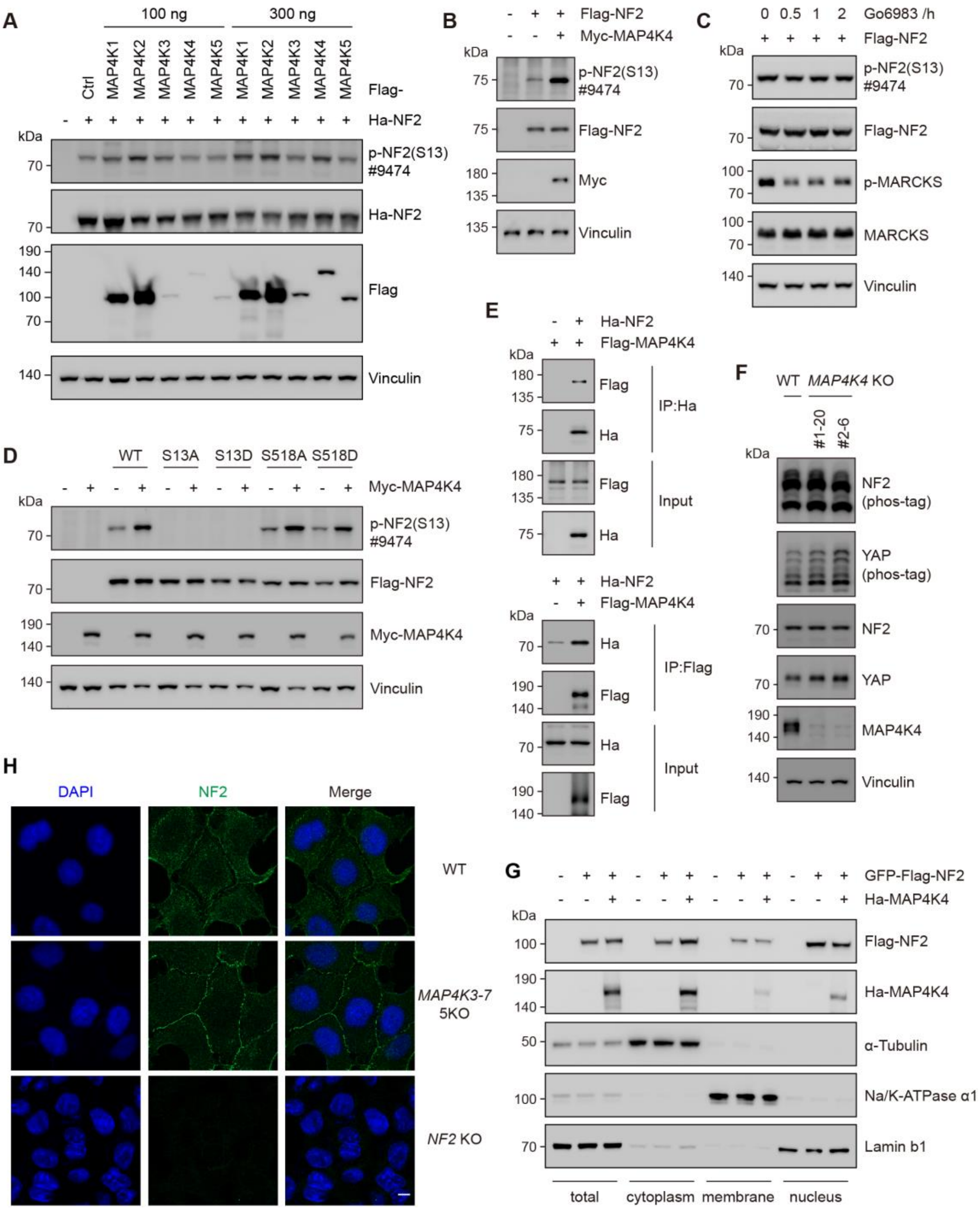

Figure S5. Regulation of NF2 phosphorylation by MAP4Ks.

(A) MAP4Ks induce NF2 phosphorylation at S13. Different kinases were transfected into HEK293A cells expressing Ha-NF2. pS13 and NF2 levels were determined.

(B) MAP4K4 induces NF2 S13 phosphorylation.

(C) PKC inhibitor does not reduce NF2 S13 phosphorylation. PKC inhibitor Go6983 (200 nM) was used to treat HEK293A cells. MARCKS is the substrate of PKC.

(D) MAP4K4 phosphorylates NF2 at S13, which is not affected by S518.

(E) MAP4K4 and NF2 interact with each other. NF2 or MAP4K4 was expressed in HEK293A cells alone or together, and coimmunoprecipitation assay was performed.
(F) *MAP4K4* deletion does not reduce NF2 S13 phosphorylation.
(G) MAP4K4 expression promotes NF2 cytoplasmic localization. In the presence of ectopic MAP4K4, cytoplasmic NF2 was increased.
(H) MAP4Ks inhibit plamsa membrane localization of NF2. Compared to WT cells, *MAP4K3-7* 5KO cells showed more NF2 membrane localization. *NF2* KO cells were used to test the specificity of NF2 antibody. Scale bar, 10 μm.

Figure S6

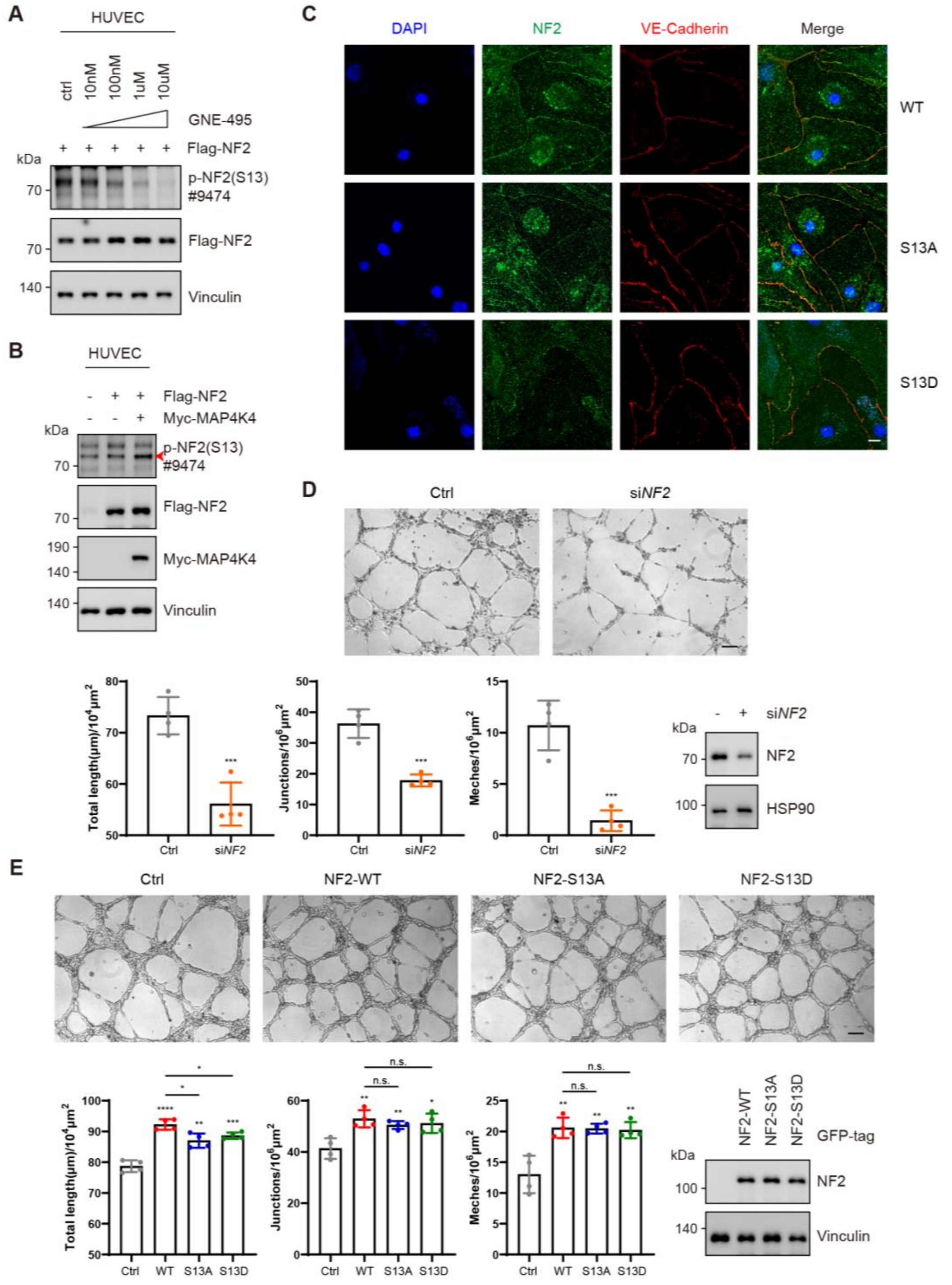

Figure S6. The function of NF2 phosphorylation in endothelial cell.

(A) GNE-495 inhibits NF2 S13 phosphorylation in HUVEC cells. MAP4Ks inhibitor GNE-495 was used to treat HUVEC for 24 h.

(B) MAP4K4 induces NF2 phosphorylation at S13 in HUVEC cells. Arrow head indicates S13 phosphorylated NF2. The pS13 signal overlapped with non-specific bands.

(C) Subcellular localization of NF2 in mouse liver endothelial cells. The membrane localization of endogenous NF2 was reduced in S13D knockin mice. VE-Cadherin marks cell junctions of endothelial cells. Scale bar, 10 μm.

(D) Effects of *NF2* knockdown in tube formation assay. *NF2* knockdown inhibited the tube formation ability of HUVEC. Scale bar, 100 μm.

(E) NF2 and S13A/D mutants promote tube formation. NF2 WT, S13A, or S13D expression significantly improved the tube formation ability of HUVEC cells. Scale bar, 100 μm.

Figure S7

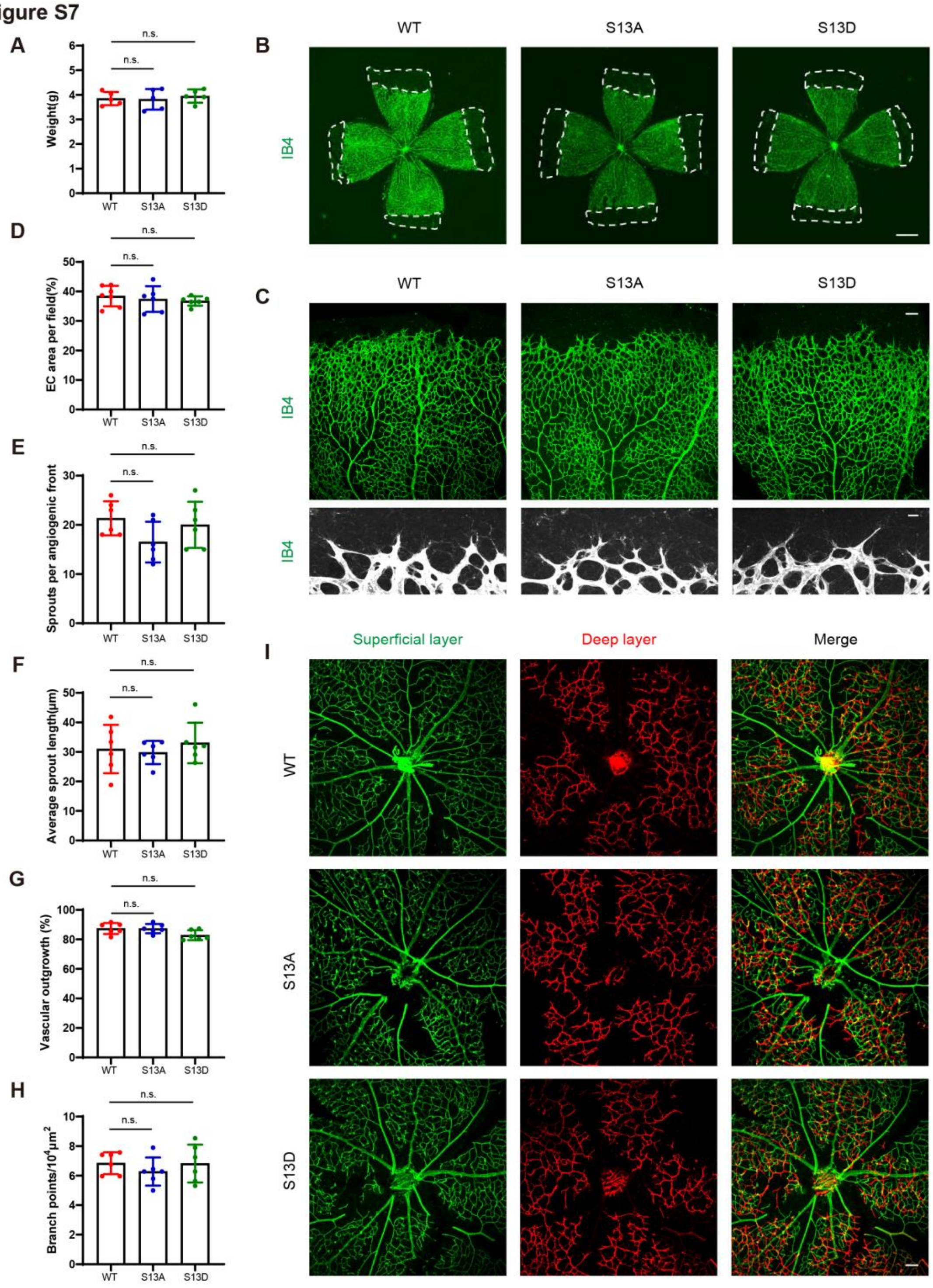

Figure S7. NF2 S13 phosphorylation has no obvious effect on developmental retinal angiogenesis.

(A) Mice used in this experiment have similar body weight.

(B) Isolectin B4 (IB4)-stained whole-mount retinal vasculature of P7 mice. Compared with WT mice, S13A and S13D mice showed no significant effect on endothelial area. Scale bar, 1 mm.

(C) Confocal images of IB4-stained retinal vasculature at P7. WT, S13A and S13D mice all showed normal sprouting (scale bar, 100 μm) and tip cell morphology (scale bar, 20 μm).

(D) Quantification of endothelial cell-covered area in the whole retinas.

(E) Quantification of sprout number per angiogenic front.

(F) Quantification of average sprout length.

(G) Quantification of vascular outgrowth.

(H) Quantification of nuber of branch points.

(I) Confocal images of IB4-stained retinal vessels at P10. No significant differences in superficial layer and deep layer retinal vasculature. Scale bar, 100 μm.
